# Patterns of Cortical Oscillations Organize Neural Activity into Whole-Brain Functional Networks Evident in the fMRI BOLD Signal

**DOI:** 10.3389/fnhum.2013.00080

**Published:** 2013-03-14

**Authors:** Jennifer C. Whitman, Lawrence M. Ward, Todd S. Woodward

**Affiliations:** ^1^Department of Psychiatry, University of British ColumbiaVancouver, BC, Canada; ^2^BC Mental Health and Addictions Research InstituteVancouver, BC, Canada; ^3^Department of Psychology, University of British ColumbiaVancouver, BC, Canada

**Keywords:** BOLD, electrophysiology, connectivity, oscillation, synchronization

## Abstract

Recent findings from electrophysiology and multimodal neuroimaging have elucidated the relationship between patterns of cortical oscillations evident in EEG/MEG and the functional brain networks evident in the BOLD signal. Much of the existing literature emphasized how high-frequency cortical oscillations are thought to coordinate neural activity locally, while low-frequency oscillations play a role in coordinating activity between more distant brain regions. However, the assignment of different frequencies to different spatial scales is an oversimplification. A more informative approach is to explore the arrangements by which these low- and high-frequency oscillations work in concert, coordinating neural activity into whole-brain functional networks. When relating such networks to the BOLD signal, we must consider how the patterns of cortical oscillations change at the same speed as cognitive states, which often last less than a second. Consequently, the slower BOLD signal may often reflect the summed neural activity of several transient network configurations. This temporal mismatch can be circumvented if we use spatial maps to assess correspondence between oscillatory networks and BOLD networks.

It is understood that whereas functional MRI (fMRI) can effectively describe the spatial activation patterns of whole-brain networks, it is limited to measuring the delayed hemodynamic response observed in the blood oxygen level dependent (BOLD) signal. This delay limits its ability to describe rapid changes in neural activity underlying the sequential processing stages inherent to any cognitive task. We can obtain much higher temporal resolution from electroencephalography (EEG), magnetoencephalography (MEG), or electrocorticography (ECoG) data. However, due to limitations on the number and location of feasible electrode placements and to difficulties in measuring signals from sub-cortical regions, these methods do not provide complete whole-brain neural activity measures with a spatial precision equivalent to that of fMRI. The above temporal and spatial limitations can be addressed through studies combining fMRI data with simultaneously recorded EEG data, or with co-registered MEG or ECoG data recorded in separate sessions. To best interpret the results of such multimodal studies, one must understand the relationship between the BOLD signal, neuronal activity at a local level, oscillations in neuronal assemblies, and the large-scale rapid oscillatory networks measurable with EEG, MEG, and ECoG. Here, we describe how cortical oscillations organize post-synaptic potentials and neuronal firing, functionally connecting activity in disparate regions to form the widespread cortical networks observed in the BOLD signal.

## Neuronal Ensembles and Local Metabolic Demand: Coordinated Neural Activity and Oscillations

At the local level, several studies have used invasive techniques to simultaneously record action potentials (APs), BOLD signal strength, and cortical oscillations. It should be noted that the BOLD signal might be expected to correspond less closely to APs measured via single-cell and multi-unit recordings than to local field potentials (LFPs), which reflect post-synaptic potentials summed across large numbers of neurons, because a single fMRI voxel typically contains more than a million neurons (Arthurs and Boniface, 2002). Indeed, the BOLD signal is reported to correlate more closely with LFPs than with APs (Logothetis et al., 2001; Arthurs and Boniface, 2002; Mukamel et al., 2005; Niessing et al., 2005). More specifically, the BOLD signal tends to correspond closely to LFPs oscillating in the high gamma range (∼50–100 Hz) although the precise range can vary across studies (Logothetis et al., 2001; Arthurs and Boniface, 2002; Mukamel et al., 2005; Niessing et al., 2005; Nir et al., 2007). However, the BOLD signal is coupled to neuronal firing when the firing rates of neighboring neurons (smoothed across periods of a few 100 ms) are most highly correlated. This coupling between firing and BOLD can depend on whether the type of visual stimulus being presented is an optimal driver of the neurons (Lippert et al., 2010). These periods also involve strong coupling between neuronal firing and gamma-frequency oscillatory power (Nir et al., 2007). This suggests that, at the local level, the BOLD signal may reflect the coordination of neuronal firing mediated by gamma-frequency oscillations in post-synaptic potentials. These oscillations could facilitate communication between neurons by ensuring that spikes arrive at the moment of peak excitability (Fries, 2005).

Gamma-frequency oscillations in the post-synaptic potentials summed across many neurons are believed to integrate activity between spatially disparate neuronal ensembles. Phase-locking of gamma oscillations is reported to coordinate activity between visual cortical neurons responding to different parts of the same moving object (Gray and Singer, 1989; Gray et al., 1989). Distinct sub-frequencies of gamma oscillations are thought to coordinate activity across distinct sets of regions in the hippocampus and parahippocampal cortices. Medium-frequency gamma (∼60 Hz) appears to coordinate activity between CA1 hippocampal neurons (deep layer) and medial entorhinal cortex. Low-frequency gamma (∼40 Hz) appears to coordinate activity between CA1 hippocampal neurons (superficial layer) and CA3 hippocampal neurons (Colgin et al., 2009; Belluscio et al., 2012). While these gamma oscillations coordinate activity between nearby regions, they also play a role in segregating neuronal ensembles involving different layers of CA1. Further evidence of the role of gamma oscillations in segregation involves “inhibitory sculpting” constraining activity to within individual cortical columns (Contreras and Llinas, 2001). In sum, these findings suggest that gamma oscillations dynamically combine local elements into neuronal ensembles while keeping the ensembles distinct from each other.

## Orchestration: Networks Formed from Multiple Frequencies of Oscillations

While gamma oscillations are effective in combining activity at the local level, it has been suggested that slower oscillations would be more optimal for coordinating activity across longer distances between hemispheres or between frontal and posterior regions, which can involve longer, poly-synaptic conduction delays of dozens of milliseconds (von Stein and Sarnthein, 2000; Varela et al., 2001). Whereas this is consistent with findings of local synchronization in the gamma-band, more long-range task-induced synchronization of slower alpha (∼10 Hz) oscillations between occipital and parietal regions, and frontoparietal synchronization in the theta band (Gray and Singer, 1989; Gray et al., 1989; von Stein and Sarnthein, 2000; Colgin et al., 2009; Doesburg et al., 2009a), there are also reports of long-distance EEG gamma-band synchronization between electrodes over frontal and parietal/occipital cortices (Doesburg et al., 2008). Furthermore, gamma-frequency LFPs are reported to correlate with BOLD signal in distant cortical regions (Schölvinck et al., 2010). Thus, spatial coordination cannot be simplified to a linear relationship between oscillatory frequency and the distance over which information is organized.

The short-range and long-range organization of neural activity by oscillations of different frequencies depends upon the ways in which those oscillations work in concert. One means by which oscillations are widely reported to work in concert involves the gamma and theta frequency ranges. The amplitude of gamma oscillations can be modulated by the phase of a theta oscillation (Canolty et al., 2006; Jensen and Colgin, 2007; Doesburg et al., 2009b). The amplitude of broadband high gamma activity (80–150 Hz) is maximal at the trough of 5 Hz theta oscillations in ECoG data collected from a wide region of cortex (Canolty et al., 2006).

This pattern of theta-modulated gamma appears to play an important role in cognitive processing. During binocular rivalry, it connects a frontoparietal network involved in perceptual switching. Immediately prior to a perceptual switch and concomitant response, it dynamically connects inferior temporal and motor cortices to the network (Doesburg et al., 2009b). It has been postulated that the timing of gamma oscillations within a given theta cycle plays an organizational role, with each 40 Hz gamma cycle within a 7 Hz theta cycle corresponding to the neuronal ensemble for one item in a set maintained in working memory (Lisman and Idiart, 1995; Jensen and Colgin, 2007). While empirical data remain inconclusive regarding that particular model of working memory, there is evidence that theta phase distinguishes between neuronal ensembles oscillating at different gamma frequencies. In rats exploring a maze, medium-frequency (50–90 Hz) gamma oscillations in the hippocampus occur at a slightly earlier phase of the theta cycle than slower (30–50 Hz) gamma oscillations (Belluscio et al., 2012). As these different gamma frequencies correspond to different neuronal ensembles, as described in the previous section, this demonstrates how phase-amplitude modulation serves to distinguish between ensembles in neighboring regions.

In addition to modulating gamma amplitude, theta phase can also modulate gamma phase. Cross-frequency theta-gamma phase–phase coupling occurs in the hippocampus and parahippocampal cortex in the medium (50–90 Hz) and slow (30–50 Hz) gamma ranges (Belluscio et al., 2012). Theta phase can also alter synchrony between gamma oscillations originating in disparate regions. Prior to a change in percept in a binocular rivalry paradigm, both intra-regional gamma-band synchronization and inter-regional synchronization between frontal and posterior regions increased. Both types of synchronization were also modulated at the theta frequency (Doesburg et al., 2009b). Thus, gamma oscillations can be phase-locked over long distances, but slower oscillations tend to be the mechanism by which local ensembles oscillating within the gamma range are organized into networks and sub-networks.

## Correspondence between Oscillatory Networks and fMRI Networks

The precise mechanism by which oscillations form functional networks varies with task demands and with the specific cortical regions involved in a given network. If we consider the regions of the dorsal and ventral attention networks often reported in the fMRI literature (Fox et al., 2005; Corbetta et al., 2008), we can find corresponding patterns of oscillatory connectivity at a range of frequencies. Alpha (∼10 Hz) oscillations are phase-locked between the superior parietal lobule (SPL) and inferior occipital gyrus (IOG) contralateral to attended locations (Doesburg et al., 2009a). Beta (∼20 Hz) oscillations are reported to synchronize activity between the frontal eye fields (FEF), the intraparietal sulcus (IPS), and occipitotemporal cortices (Hipp et al., 2011). Theta and gamma oscillations are reported to synchronize frontal regions such as the dorsolateral prefrontal cortex (DLPFC) and superior frontal gyrus (SFG) to the precentral gyrus and precuneus (Doesburg et al., 2009b). Slower oscillations (slow cortical potentials or “up/down” states) in EEG/MEG/ECoG data occur at a rate comparable to the spontaneous resting-state oscillations measurable in the BOLD signal (He et al., 2008; He and Raichle, 2009). In sum, power in no single frequency band can be said to be the signal driving the BOLD response. Instead, several different frequencies of oscillation will likely work in concert to connect the regions belonging to a given fMRI network, particularly if that network involves many regions. This view would be consistent with recent findings that the amplitude and time-course of the hemodynamic response are dependent not only on gamma-frequency power (although gamma power is the strongest predictor of BOLD signal amplitude) but also alpha and beta power (Magri et al., 2012). It is also consistent with recent findings that coherent low-frequency oscillations were the predominant contributors to inter-regional correlations in BOLD signal in a thalamo-cortical visual network. These slow oscillations modulated local high-frequency (gamma) activity via cross-frequency coupling (Wang et al., 2012).

The involvement of different oscillatory frequencies in a cortical network varies as a function of cognitive state (von Stein and Sarnthein, 2000; Doesburg et al., 2008, 2009a,b; Brookes et al., 2011), and those states often last only a few 100 ms. Given that the hemodynamic response is delayed by several seconds, fMRI cannot distinguish between the neural responses to brief sequential cognitive states. Thus, a given fMRI network configuration might reflect the sum of several oscillatory network configurations, each of which had a brief duration. When interpreting the results of multimodal studies, researchers should expect that each network identified in their fMRI data is likely to decompose into multiple oscillatory network configurations. In addition, they should expect fMRI data reflecting early sensory processing to correspond most closely to evoked responses in EEG/MEG/ECoG data, as much of signal contributing to the event-related averages in these data involves oscillations phase-locked to the onset of sensory stimuli. In contrast, fMRI data reflecting high-level cognitive processing involving cognitive stages with more variable timing should correspond more closely to induced responses in EEG/MEG/ECoG data, as these reflect oscillations not precisely phase-locked to the onset of sensory stimuli. It should be noted that both evoked and induced responses may derive from frequency-dependent changes in phase alignment (Burgess, 2012).

## Directions for Future Research

Much of the work remaining for future research involves describing cognitive processes in terms of the activities of their underlying brain networks, identifiable both in BOLD signals and in patterns of electrophysiological activity. The combined high spatial and temporal resolution of multimodal analyses, as well as the potential for identifying complex multi-frequency oscillatory patterns, represents an opportunity for extensive discovery by cognitive neuroscientists. A promising approach to combining the spatial resolution of fMRI with the temporal resolution of EEG or MEG involves identifying oscillatory networks with high spatial correspondence to fMRI networks. The time-course of oscillatory activity can then be described with high temporal resolution, while the fMRI data provide high confidence in the locations of the generators of the oscillatory signals.

There is also a need for more basic research on the generating mechanisms of neuronal oscillations (Burns et al., 2011), extending our understanding beyond the hippocampus to a diversity of neocortical and sub-cortical regions. One interesting finding in this area involves patterns of oscillatory connectivity between neocortical layers. Oscillatory activity in V1 appears to be compartmentalized within either infragranular layers, which project largely to thalamic regions, or granular and supragranular layers, which project mainly to other cortical regions (Maier et al., 2010). It would be interesting to investigate whether communication between those compartments was accomplished via the cross-frequency coupling underlying connectivity in thalamo-cortical visual networks (Wang et al., 2012). Finally, understanding how the brain networks evident in multimodal studies are affected by genetic variants and neurotransmitter levels will be integral to understanding clinical etiology and advancing treatment.

## Conclusion

If multimodal studies attempt to identify the electrophysiological metric that best predicts the BOLD signal, they will produce findings of limited generalizability. There is no single oscillatory frequency range, and no single measure of neuronal oscillation or synchronization, that can be said to be the best predictor. Rather, the pattern of correspondence between electrophysiology and hemodynamics will depend upon whether one compares spatial patterns of network activity or network time courses. It will also depend upon whether one compares signal strength at a local level or at a whole-brain level. Finally, it will depend upon whether data are recorded at rest or during performance of a cognitive task, and upon the specific cognitive demands of said task. Converging results obtained using diverse measurements suggest that the BOLD signal strength corresponds well with high-frequency oscillatory power at the local level, and that functional connectivity in the BOLD signal across greater distances corresponds well with lower frequency oscillations. It would be more fruitful, however, for researchers to explore how these different oscillatory frequencies work in concert. A particular pattern representing a combination of low and high frequencies (such as when high-frequency amplitude depends upon low-frequency phase) may organize activity at a local level by integrating some signals while segregating others, and simultaneously coordinate multiple organized local patterns across much greater distances. These multi-frequency patterns provide an optimal explanation for the mechanisms of cognitive processing because they dynamically change at the same pace as cognition.

## Conflict of Interest Statement

The authors declare that the research was conducted in the absence of any commercial or financial relationships that could be construed as a potential conflict of interest.
